# Validation of Reference Genes for Quantitative Expression Analysis by Real-Time RT-PCR in Four Lepidopteran Insects

**DOI:** 10.1673/031.012.6001

**Published:** 2012-05-09

**Authors:** Xiaolu Teng, Zan Zhang, Guiling He, Liwen Yang, Fei Li

**Affiliations:** ^1^ Department of Entomology, College of Plant Protection, Nanjing Agricultural University, Nanjing, 210095, China; ^2^ Key Laboratory of Integrated Management of Crop Diseases and Pests, Nanjing Agricultural University, Ministry of Education, 210095, China; ^3^ Department of Chemistry, Jiaying University, Meizhou, Guangzhou, 514015, China

**Keywords:** expression stability, housekeeping gene, qPCR, reference gene

## Abstract

Quantitative real-time polymerase chain reaction (qPCR) is an efficient and widely used technique to monitor gene expression. Housekeeping genes (HKGs) are often empirically selected as the reference genes for data normalization. However, the suitability of HKGs used as the reference genes has been seldom validated. Here, six HKGs were chosen (*actin A3*, *actin A1*, *GAPDH*, *G3PDH*, *E2F*, *rp49*) in four lepidopteran insects *Bombyx mori* L. (Lepidoptera: Bombycidae), *Plutella xylostella* L. (Plutellidae), *Chilo suppressalis* Walker (Crambidae), and *Spodoptera exigua* Hübner (Noctuidae) to study their expression stability. The algorithms of geNorm, NormFinder, stability index, and ΔCt analysis were used to evaluate these HKGs. Across different developmental stages, *actin A1* was the most stable in *P. xylostella* and *C. suppressalis*, but it was the least stable in *B. mori* and *S. exigua. Rp49* and *GAPDH* were the most stable in *B. mori* and *S. exigua*, respectively. In different tissues, *GAPDH*, *E2F*, and *Rp49* were the most stable in *B. mori*, *S. exigua*, and *C. suppressalis*, respectively. The relative abundances of *Siwi* genes estimated by 2^-ΔΔCt^ method were tested with different HKGs as the reference gene, proving the importance of internal controls in qPCR data analysis. The results not only presented a list of suitable reference genes in four lepidopteran insects, but also proved that the expression stabilities of HKGs were different among evolutionarily close species. There was no single universal reference gene that could be used in all situations. It is indispensable to validate the expression of HKGs before using them as the internal control in qPCR.

## Introduction

Estimating transcript expression levels across different conditions is an important aspect of work in molecular biology. Many techniques such as northern blot, microarray, and quantitative real-time polymerase chain reaction (qPCR) have been developed to determine gene expression levels. Among these, qPCR is one of the best choices and is widely applied in monitoring transcript-level changes in gene expression.

In several steps of the qPCR protocol, including preparing material, RNA purification, cDNA synthesis, and PCR procedures, non-specific variations may be introduced. For example, the efficiencies of reverse transcription (RT) and PCR may be varied between samples. Therefore, selection of one or more reference genes, also called internal controls, is very important to eliminate the inter-sample variations in analyzing qPCR data. An ideal reference gene is constantly expressed in all samples under certain condition. Housekeeping genes (HKGs) are thought to have key roles in cellular processes and are expressed at a constant level, and thus are often used as reference genes in qPCR. There are two strategies to choose a reference gene. One is to use HKGs as reference genes that have already used in previous studies; another is to use HKGs that are homologous to a widely used reference gene in other model species.

These empirical choices often lack experimental support. Increasing evidence has suggested that HKGs are not always expressed stably in all experimental conditions. The α-*actin* gene, which has been widely used as a reference gene, was reported to be highly regulated by matrigel, and therefore unsuitable as an internal control in this condition (Selvey et al. 2001). Several housekeeping genes, such as α-tubulin and *GAPDH*, were also reported to have varied expression levels in some instances such as different tissues or different developmental stages (Brunner et al. 2004; Trivedi and Arasu 2005; Pei et al. 2007; Dhar et al. 2009; Dong et al. 2010; Lord et al. 2010; Wang et al. 2010). Some studies also proved that choosing unsuitable reference genes produced low precision or misleading results (Jian et al. 2008). Therefore, it is necessary to validate the expression stabilities of HKGs when choosing them as reference genes in qPCR.

Though qPCR has been widely used to estimate the gene expression in insects, very few studies have been performed to validate the expression stability of HKGs. In *Bombyx mori* L. (Lepidoptera: Bombycidae), translation initiation factor 4A, translation initiation factor 3 subunit 4, and translation initiation factor 3 subunit 5 were reported to be the most reliable reference genes during metamorphosis (Wang et al. 2008). However, the most commonly used reference gene, cytoplasmic actin, varied drastically throughout metamorphosis development (Wang et al. 2008). Ribosomal protein genes RPS3, RPS18, and RPL13a were the most stable genes in *Tribolium castaneum* exposed to *Beauveria bassiana* (Lord et al. 2010). Here, four economic and agriculturally important lepidopteran insects were chosen, including *B. mori*, *Plutella xylostella* L. (Plutellidae), *Chilo suppressalis* Walker (Pyralididae), and *Spodoptera exigua* Hübner (Noctuidae) to evaluate the expression stability of six HKGs (*actin A3*, *actin A1*, *GAPDH*, *G3PDH*, *E2F*, and *rp49*). Reliable reference genes are proposed in these four lepidopteran insects.

## Materials and Methods

### Sample collection

All insects were maintained at 25 ± 1 °C under a photoperiod of 16:8 L:D and 80% RH. The silkworms (*B. mori*) were fed on mulberry leaves, diamondback moths (*P. xylostella*) were reared on radishes, rice stem borers (*C. suppressalis*) were fed on rice seedling, and beet armyworms (*S. exigua*) were fed on an artificial diet (Huang et al. 2002; Merkx-Jacques and Bede 2005). The different developmental stages collected were egg, larvae (collected at the first day of each instar), pupa, and adult, for each insect. Tissues from the head, midgut, ovary, testis, fat body, malpighian tube, and epidermis were dissected from final instar larvae and kept at -70 °C for RNA purification. The silk glands of the *B. mori* were also collected for experiments. Two sets of samples were collected for experimental replication. About 30 insects were collected for each sample, and all experiments for each set of samples were repeated three times.

### Collecting the sequences of candidate reference genes

A list of housekeeping genes (HKGs) was compiled that have been commonly used as the internal controls for normalization in qPCR data analysis in four insects by reference mining. The sequences of selected HKGs of *P. xylostella*, *C. suppressalis*, and *S. exigua* were obtained by searching transcriptome data. The sequences of *B. mori* genes were downloaded from the refseq database of NCBI. All the primers were designed using Beacon Designer 7 (Premier Biosoft, www.premierbiosoft.com). The sequences of primers are given in Table 1. *Siwi*-1 and *Siwi*-2 genes were chosen to study the impact of reference genes on the qPCR data analysis. The forward primer sequence of *Siwi*-1 was 5′- GTGCGGGTCTGCCTGAGTTG-3′ and the reverse primer sequence of *Siwi*-1 was 5′GGTGTGCTTGTGAATCTCCTGTTG-3′. The forward primer sequence of *Siwi*-2 was 5′-AGCCTACCCTGACCTATGTTG-3′ and the reverse primer sequence of *Siwi*-2 was 5′ACCAGTCCCGTCTCGTTATAC-3′.

### RNA purification

The samples were frozen with liquid nitrogen and homogenized in a tissue grinder. Then, 1 mL TRIzol reagent (GIBCO, www.invitrogen.com) was added to homogenized samples. Total RNA was isolated following the recommended procedures. Genomic DNA was removed from total RNAs by DNase I treatments following the protocol of the DNA-free kit (Ambion, www.invitrogen.com). The integrity of RNA was checked on a 1.5% agarose gel and visualized by ethidium bromide staining. The concentrations and quality of RNA were also measured with Nanodrop ND-1OOO (Thermo Scientific, www.thermoscientific.com). The 260/280 nm absorbance ratios of all RNA samples were between 1.8 and 2.2. First strand cDNA was synthesized from 1 µg total RNA using MMLV reverse transcriptase (Takara Bio Inc., www.takara-bio.com) and Oligo (dT18) as the anchor primer. The reaction mixtures were incubated at 70 °C for 10 min followed by 42 ° C for one hour and 70 °C for 15 min.

### Quantitative real-time PCR

The qPCR reactions were carried out with SYBR Premix Ex Taq (Takara Bio Inc.) following the manufacturer's protocol using an ABI Prism 7300 (Applied Biosystems, www.appliedbiosystems.com). The reaction mixture consisted of 2 µL of cDNA template in a final reaction volume of 20 µL. The qPCR protocol included an initial step of 95 for 30 sec, followed by 40 cycles of 95 °C for five sec and then annealed at 60 °C for 31 sec, followed by one cycle of 95 °C for 15 sec, 60 ° C for 60 sec, and 95 °C for 15 sec. Products were dissolved by curve analysis (60-95 °C) after 40 cycles. The specificity of the qPCR reactions was monitored with melting curve analysis using SDS software (version 1.4) and gel electrophoresis. Amplification efficiencies were determined by a series of template dilutions. All experiments were repeated in triplicate.

### Data processing

The raw Ct values were obtained using the SDS software of ABI 7300 (version 1.4). The algorithms including geNorm (Vandesompele et al. 2002), Normfinder (Andersen et al. 2004), ΔCt approach (Silver et al. 2006), and Stability index (Brunner et al. 2004) were used to analyze the raw Ct values of selected HKGs. The analysis procedures strictly followed the manuals of the algorithms. The fold changes of *Siwi*-1 and *Siwi*-2 genes in different developmental stages or tissues were calculated using standard delta-delta-Ct method (Pfaffl 2001).

## Results

### Identification of candidate housekeeping genes

According to the mining of qPCR publications, six commonly-used candidate HKGs were selected to validate their expression stability, including cytoplasmic *actin* gene A3 (*actin A3*), cytoplasmic *actin* gene A1 (*actin A1*), glyceraldehyde-3-phosphate dehydrogenase (*GAPDH*), glycerol-3-phosphate dehydrogenase-2 (*G3PDH*), *E2F* transcription factor 4 (*E2F*), and ribosomal protein 49 (*rp49*). The sequences of six HKGs in *B. mori* were obtained by searching the refseq database of NCBI (Walter et al. 2009). The HKGs of three lepidopteran pests, *C. suppressalis*, *P. xylostella*, and *S. exigua* were searched from the transcriptome data using the BLASTX program (unpublished data). The sequences of *actin A3* in *B. mori* were used to search for homologs in the three lepidopteran pests, but homologs were not found and thus not used in further experiments. *Rp49* of *S. exigua* was not considered for further analysis because it had a very short sequence of poor quality.

Primer specificities were confirmed by melting curve analysis and all primer pairs amplified a single PCR product with the expected sizes. PCR products were confirmed by bi-direction sequencing. Amplification efficiencies of primers reached the standard requirements of conventional qPCR, which were determined by a serial dilution of cDNA template. All the experiments including RNA purification, cDNA synthesis, and qPCR were carried out by the same individual using the same reagent lot.

### Expression levels of the reference gene candidates

**Figure 1.**
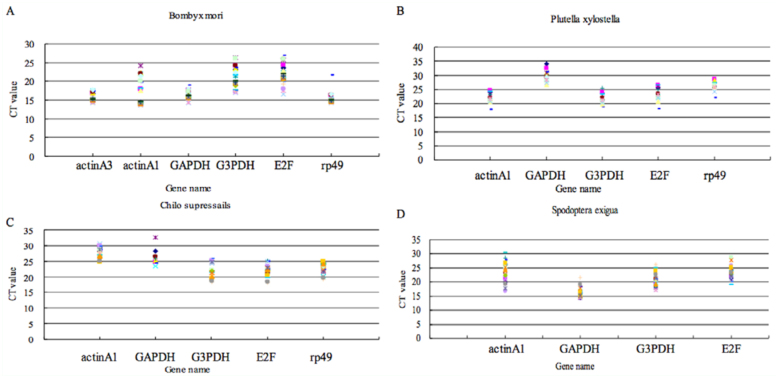
The variance of raw Ct values of selected housekeeping genes (HKGs) in all tested samples. High quality figures are available online.

The Cycle threshold (Ct) values in qPCR gave an overview of the gene expression variation in the samples. All selected HKGs had moderate abundance in different developmental stages of *C. suppressalis.* The mean Ct values of HKGs in four insect species were 15.20–29.56 cycles. Individual HKGs had different expression levels across all tested samples. According to the variations of Ct values, *GAPDH* showed the smallest gene expression variation (below five cycles) in *B. mori* and *S. exigua*, while *actin A1* had the highest expression variation (above eight cycles). However, *actin A1* was the most stable gene *C. suppressalisand P. xylostella*, whereas *GAPDH* was the least stable. The wide range of Ct values of six tested HKGs indicated that there was no HKG with constant expression under these conditions (Figure 1). Thus, determining a suitable HKG as a reference gene requires careful confirmation of expression stability.

### Expression stability of selected HKGs across different developmental stages

**Supplementary Figure 1.**
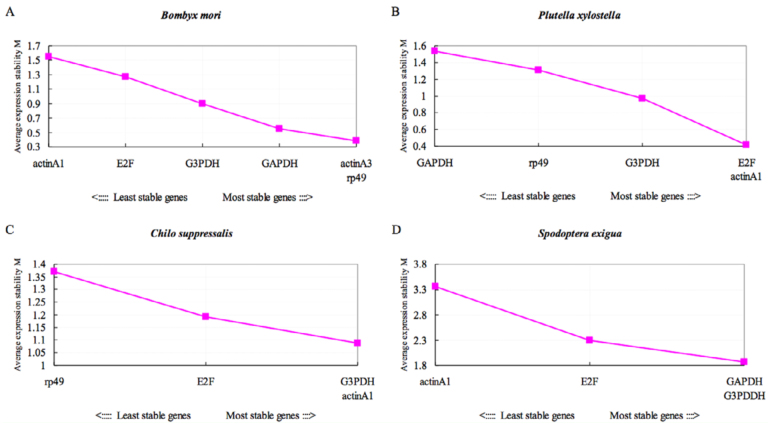
The expression stability of selected housekeeping genes (HKGs) across different developmental stages estimated by geNorm. High quality figures are available online.

Gene expression patterns across different developmental stages are frequently investigated. Insects were collected from developmental stages including egg, larvae collected at the first day of each instar, pupa, and adult, in order to study the expression stability of selected HKGs. Raw Ct values were first transformed to relative quantities using the ΔCt method. Then, geNorm and NormFinder were used to calculate the expression stability of selected HKGs. geNorm estimates the gene expression stability by calculating stability measure *M* for an HKG as the average pairwise variation for that gene with all other tested HKGs. The HKGs with low *M* values are the stably expressed genes. Based on this principle, geNorm recommend a pair of the most stable HKGs as candidate reference genes (Vandesompele et al. 2002). According to the results of geNorm, the stabilities of selected HKGs were *actin* A3 = *rp49* > *GAPDH* > *G3PDH* > *E2F* > *actin A1* in *B. mori.* The HKG stabilities in *P. xylostella* were *Actin* A1 = *E2F* > *G3PDH* > *rp49* > *GAPDH*; in *C. suppressalis actin* A1 = *G3PDH* > *E2F* > *rp49*; in *S. exigua GAPDH* = *G3PDH* > *E2F* > *actin* A1. These results demonstrated that actin A1 was most stable in *P. xylostella* and *C. suppressalis*, whereas it was least stable in the other two lepidopteran insects across different developmental stages. *GAPDH* was the most stable HKG in *S. exigua*, whereas it was the least stable in *P. xylostella* (Table 2 and Figure S1). These results proved that the stabilities of HKGs are quite different in the four lepidopteran insects used in this study.

To confirm the results of geNorm, NormFinder was used to analyze the qPCR data. In general, the optimum reference genes found by NormFinder were similar to those of geNorm, suggesting the reliability of data analysis. Both geNorm and NormFinder ranked *actin* A1 as the most stable HKG in *P.* *xylostella* but the least stable in the *B. mori* and *S. exigua.* The least stable HKGs found by both two algorithms were the same. There was an inconsistency in determining the most stable gene in *B. mori* and *C. suppressalis* by two algorithms. In *B. mori*, *GAPDH* was evaluated as the most stable gene by NormFinder, but *rp49* and *actin A3* were the best candidates for reference genes by geNorm. In *C. suppressalis*, *E2F* was the most stable by NormFinder whereas *actin A1* and *G3PDH* were the most stable HKGs (Table 2). Taking all factors into account, we recommend that *rp49* in the *B. mori*, *actin A1* in *P. xylostella* and *C. suppressalis*, and *GAPDH* in *S. exigua* be used as the reference genes across different developmental stages.

**Figure 2.**
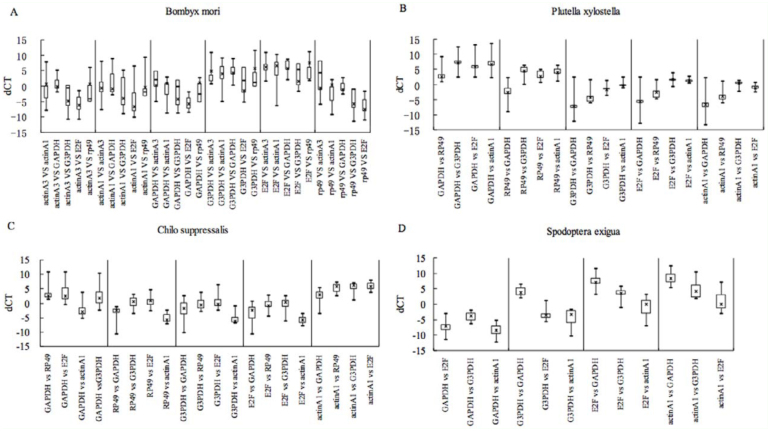
ΔCt approach analysis of expression stability of selected housekeeping gene (HKGs) in the (A) *Bombyx mori*, (B) *Plutella xylostella*, (C) *Chilo suppressalis*, and (D) *Spodoptera exigua.* The qPCR data from all tested sample were put together for analysis. ΔCt variability of HKGs is shown as medians (lines), 25^th^ percentile to the 75^th^ percentile (boxes), and ranges (whiskers) estimated from the data of all samples. High quality figures are available online.

In some cases, using the two HKGs in combination is more accurate than just using one reference gene. NormFinder not only finds the optimum reference genes out of a group of candidate genes, but also (in contrast to geNorm) takes information of groupings of samples into account (Andersen et al. 2004). Therefore, NormFinder is able to estimate the variation between sample groups, which can determine the best combination of two reference genes for normalization. The best combination of HKGs was *GAPDH* and *G3PDH* in *B. mori* with stability value of 0.421. The combinations of *actin* A1 and *G3PDH* were estimated as the best pair of reference genes in both *P. xylostella* and *S. exigua.* In *C. suppressalis*, *E2F* and *G3PDH* was the best combination with stability value of 0.454 (Table 3). It should be noticed that choosing the best combination considers the pairwise inter-variations and intra-variations in the grouping of samples. It does not rely on the stability value of an individual HKG. Actin A1 is the least stable HKG in the *S. exigua* when analyzed singly. However, it was estimated to be one of the best combinations (*actin* A1 and *G3PDH*) when considering multiple reference genes.

**Supplementary Figure 2.**
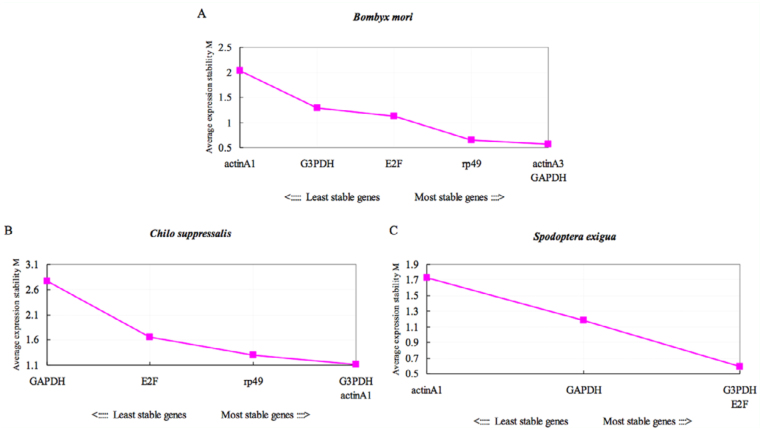
The expression stability of selected housekeeping genes (HKGs) in different tissues estimated by geNorm. High quality figures are available online.

### Expression stability of candidate reference genes in different tissues

The gene expression pattern in different tissues is also frequently studied to infer the gene functions. To screen suitable reference genes, different tissues were collected including head, midgut, ovary, testis, fat body, malpighian tube, epidermis, or silk gland of *B. mori*, *C. suppressalis*, and *S. exigua.* The expression stabilities of selected HKGs were analyzed with geNorm and NormFinder. Though there were differences between the ranks of HKGs by two algorithms, the most stable and the least stable HKGs in different tissues of three insects found by NormFinder were identical with those found by geNorm. Considering all factors, *GAPDH* was the most stable of HKGs in *B. mori*, while *actin A1* in *C. suppressalis* and *E2F* in *S. exigua* were the best candidate reference genes (Table 4 and Figure S2). In *C. suppressalis*, the expression of *actin A1* was stable among different developmental stages and tissues. However, the most stable HKGs in the different tissues of *B. mori* and *S. exigua* were not same as those across different developmental stages, suggesting that HKGs have varied expression levels under different conditions.

For the best combinations of two HKGs, *GAPDH* and *G3PDH* were the most suitable candidates in different tissues of *B. mori*, which was identical with those across different developmental stages. *Rp49* and *actin A1* in *C. suppressalis* and *GAPDH* and *E2F* in *S. exigua* were the best combinations in different tissues, which was different from those across different developmental stages (Table 3).

### Expression stability of candidate reference genes in all tested samples tissues

It is impossible to evaluate the expressions of HKGs under all kinds of experimental situations. To provide a general understanding of the expression stability of selected HKGs, the qPCR data from all tested samples was put together for evaluation. According to the results of geNorm and NormFinder, the most stable gene in *B. mori* was *GAPDH* followed by *actin A3. Actin A1* was the least stable HKG in *B. mori* and *S. exigua*, but it was the most suitable candidate of reference genes in *C. suppressalis* and *P. xylostella. GAPDH* was the most stable HKG in *S. exigua*, followed by *G3PDH* (Table 5).

To validate the analysis results of geNorm and NormFinder, stability index assay and ΔCt analysis were also used to estimate the expression variations of HKGs in all tested samples. The stability index assay was first used to select suitable internal controls during the development of poplar (Brunner et al. 2004). According to the stability index assay, *rp49* had the lowest stability index in *B. mori*, *P. xylostella*, and *C. suppressalis*, while *GAPDH* was the most stable HKG in *S. exigua* (Table 5 and Table S1). In general, stability index assay had different results with those of geNorm and NormFinder in identifying the most stable HKGs. However, the most stable HKGs found by geNorm and NormFinder also had a low stability index.

The ΔCt approach was employed to select the most suitable HKG in reticulocytes (Silver et al. 2006). The principle of the ΔCt approach was to examine the ΔCt value between the two HKGs in different samples. If the ΔCt value remains constant, it suggests that either both HKGs are stably expressed among the tested samples or are co-regulated. However, the fluctuation of the ΔCt value indicates that at least one of them is variably expressed. After introducing more HKGs into the comparisons, an appropriate HKG can be found for a particular experimental system (Figure 2). The most stable HKGs selected by the ΔCt approach were identical with those by geNorm and NormFinder, which were *GAPDH in B. mori* and *S. exigua* and *actin A1* in *P. xylostella* and *C. suppressalis* (Table 5 and Table S2).

### The impact of reference genes on qPCR data analysis

Based on the above analysis, we present evidence that the expression of HKGs in lepidopteran insects was not constant as expected. A stable HKG in one species did not mean that it also was expressed stably in another. Thus, it is our interest to study the impact of using “wrong” reference genes on the qPCR data analysis. The *Siwi*-1 and *Siwi*-2 genes in *B. mori* were chosen for evaluations of different HKGs. The samples from different developmental stages and six different tissues of the fifth instar larvae were used in the experiments.

For qPCR data analysis across different developmental stages, the egg stage was designated as the “calibrator sample“ (abundance set to 1X). The relative expression levels of all other samples were then calculated relative to the calibrator sample. The results indicated that using different reference genes had apparent effects on the qPCR analysis. *Rp49* was recommended to be the most suitable candidate of reference genes across different developmental stages. The relative abundance of both *Siwi*-1 and *Siwi*-2 using *rp49* as the reference gene were in general similar to those obtained when using *actin A3*, the second most stable HKG (Table 6). However, the results normalized using *rp49* were significantly different from those using *actin A1*, the least stable HKG, as the internal control. In the third instar larvae, the relative abundance of *Siwi*-1 was 0.314 when using *rp49* as the reference gene, but it changed to 0.067 when using *actin A1.* A big difference could also be observed for the *Siwi-*2 gene. The relative abundance of *Siwi*-2 normalized using *actin A1* (about 10-^4^ level) was significantly lower than those using *rp49* (about 10 ^2^ level) (Table 6).

For qPCR data analysis in different tissues, the head was selected as the “calibrator sample”. In this case, *GAPDH* was the most stable HKG and was suggested to be the best reference gene. If using *GAPDH* as the reference gene, the relative mRNA abundances of *Siwi*-1 in the ovary and testis were only 2.406 and 1.769, respectively, but they changed to 26.438 and 46.709 when using *actin A1* as the internal control, which was the least stable HKG (Table 7). For *Siwi-*2 gene, if *GAPDH* was used as the reference gene, the relative expression levels in ovary and testis were 1.214 and 2.488, respectively. But they were 13.347 and 64.792 when using *actin A1* for normalization. On the contrary, the results of *GAPDH* were similar with those of *actin A3*, the second stable HKG (Table 7).

These surprising differences demonstrate the importance of choosing a proper reference gene. If an unsuitable reference gene were used in normalizing qPCR data, incorrect results might be obtained and lead to improper conclusions.

## Discussion

qPCR has become a powerful tool for estimating gene expression levels because of its sensitivity and accuracy (Bustin 2000; Gachon et al. 2004; Wong and Medrano 2005; Nolan et al. 2006). The variations of qPCR will be unavoidably introduced by the procedures of RNA preparation, cDNA synthesis, or PCR processing. These variations can be controlled to some extent by carefully conducting the qRT-CPR experiments. The Ct values used for qPCR data analysis is determined mostly by the quantity of cDNA template (Walker 2002). The cDNA quantity is determined by the total RNA used in the first strand cDNA synthesis and enzymatic efficiency of reverse transcriptase. Therefore, there is a close relationship between RNA integrity and expression quantitation. The quality and purity of the total RNA should be stringently analyzed before use in qPCR analysis (Bustin and Nolan 2004; Fleige and Pfaffl 2006; Fleige et al. 2006). However, it is impossible to eliminate all variations by these strategies. It is necessary to use HKGs as the proper reference genes for normalization.

To be a good reference gene, an HKG should meet three criteria: first, it should have amplification efficiency similar to the target genes; second, it should have moderate expression level; and third, its expression should be stable in all test samples. Unfortunately, it is unlikely that a single universal reference gene can be found in all species or in all experimental conditions of a species (Bustin 2000). Almost all genes including HKGs are regulated by other “regulators”. It is unsafe to deduce the expression stability of an HKG based on known information of gene regulation. The *actin* gene is a widely used reference gene because it is thought to be stably expressed in all possible conditions. However, this gene has been challenged for its suitability as the internal control (Selvey et al. 2001). In our work, though *actin A1* was the most stable in *P. xylostella* and *C. suppressalis*, its expression was the least stable in *B. mori* and *S. exigua.*

Evaluation of the expression stability requires mathematical methods. Many algorithms such as geNorm (Vandesompele et al. 2002), Normfinder (Andersen et al. 2004), ΔCt approach (Silver et al. 2006), and stability index (Brunner et al. 2004) have been developed. The most stable HKG found by geNorm, NormFinder, and ΔCt approach were the same in all four lepidopteran insects. But stability index had different results. We reasoned that this approach only considers the stability of HKGs individually but the other three algorithms evaluate the stability of HKGs by analyzing its variance comparing with other HKGs in pair. Thus, the reliability of stability index is not as high as others. Although the expression stability analyzed by ΔCt approach was similar to that by geNorm and Normfinder, it is not a good choice for reference gene selection because it relies on a strict principle that the expression variance of two HKGs should be identical in all samples in all experimental conditions or cell types. In addition, the computation procedures of ΔCt approach are cumbersome (Silver et al. 2006). geNorm and NormFinder use different mathematical methods to estimate the expression stability. The results of two algorithms can be used for cross validation. geNorm is one of the reliable algorithms to search for stably expressed HKGs that have low intra group variation and non-vanishing inter-group variation (Jain et al. 2006; Exposito-Rodriguez et al. 2008; Gutierrez et al. 2008; Jian et al. 2008). Compared with the geNorm, NormFinder finds not only the most stable gene but also the best combination of two reference genes if multiple internal controls are considered in qPCR data analysis (Oelkers et al. 2008).

In summary, our work presented a number of the most stable HKGs that are suitable to be used as the reference genes in four lepidopteran insects. The results demonstrated that no single universal reference gene could be used in all experiment conditions or treatments. The most reliable approach of choosing suitable HKGs as reference genes is to validate their expression stability using algorithms such as geNorm and NormFinder.

**Table 1.**
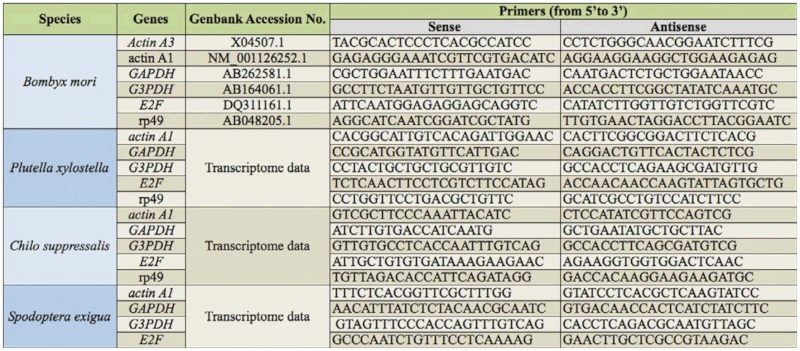
The primer sequences of selected housekeeping genes (HKGs) used in the experiments.

**Table 2.**
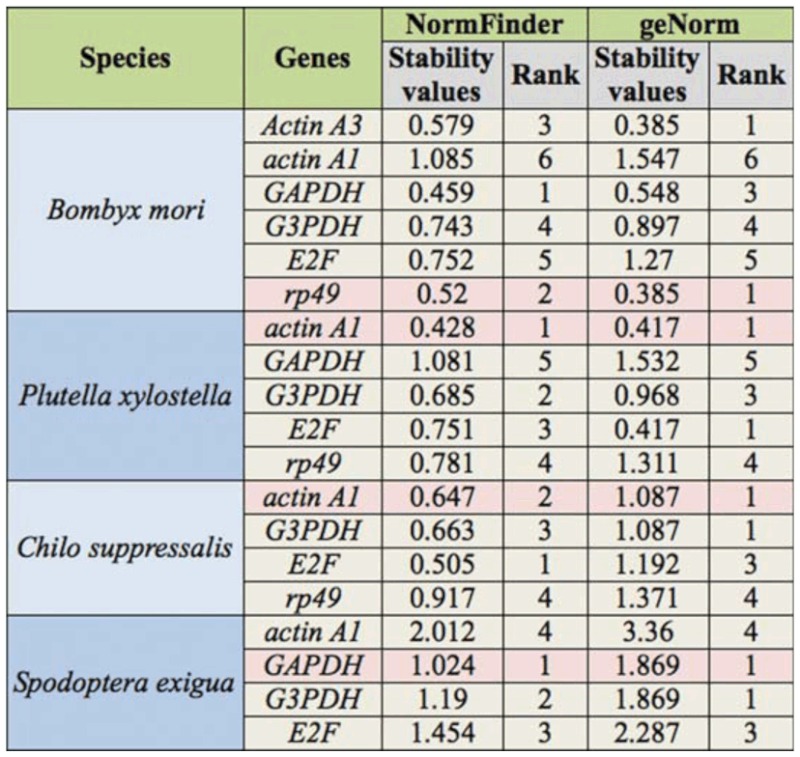
The stability values of selected housekeeping genes (HKGs) across the different developmental stages.

**Table 3.**
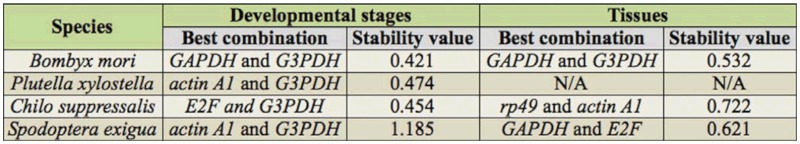
The best combination of two housekeeping genes (HKGs) recommend by NormFinder, which can be used as the multiple references genes in different developmental stages or tissues.

**Table 4.**
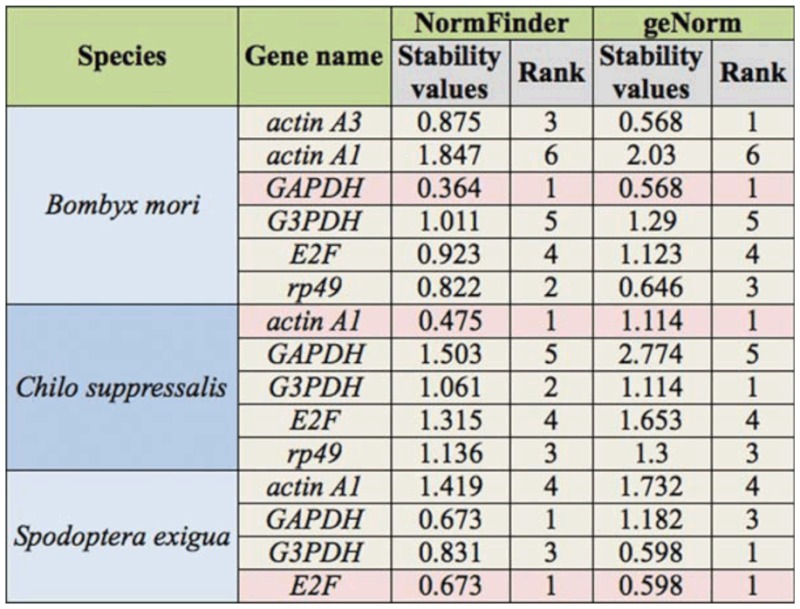
The stability values of selected housekeeping genes (HKGs) in different tissues.

**Table 5.**
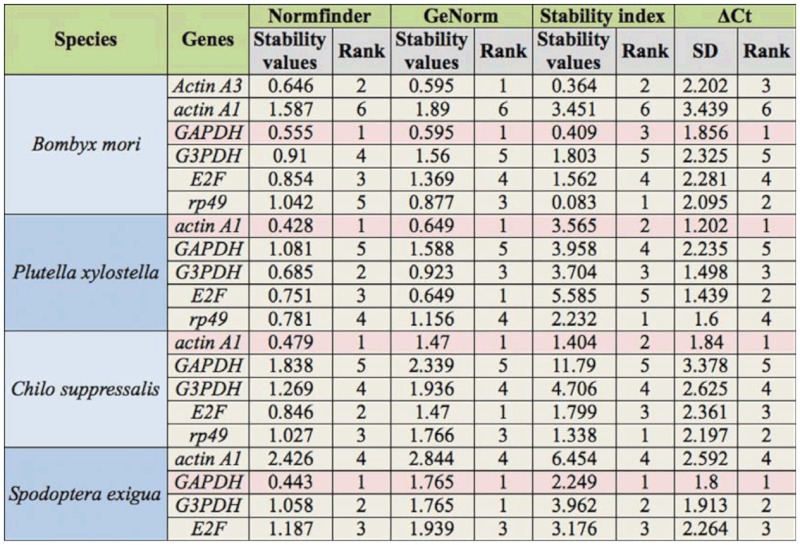
The stability values of selected housekeeping genes (HKGs) in all tested samples.

**Table 6.**
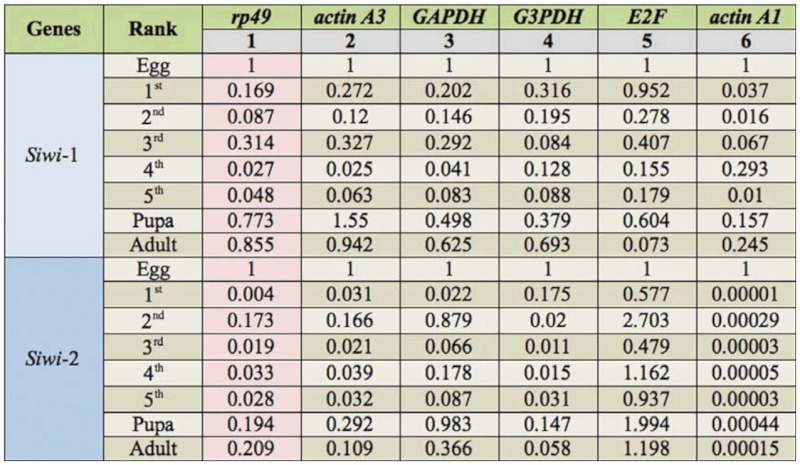
The relative mRNA abundance of *Siwi*-1 and *Siwi*-2 genes across different developmental stages of the silkworm when using different housekeeping genes (HKGs) as the internal control. The rank of reference genes was determined by geNorm.

**Table 7.**
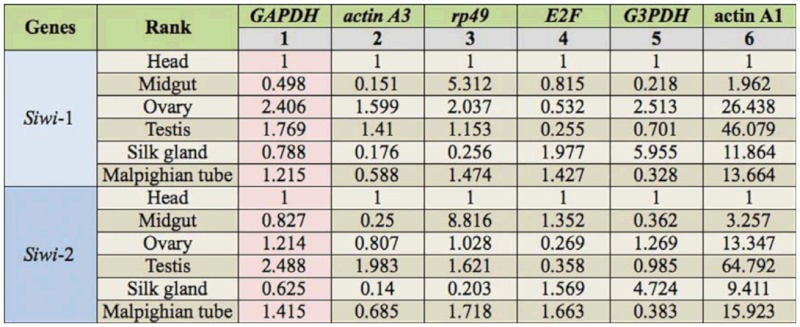
The relative mRNA abundance of *Siwi-*1 and *Siwi*-2 genes in different tissues of the silkworm when using different housekeeping genes (HKGs) as the internal control. The rank of reference genes was determined by geNorm.

**Supplementary Table 1.**
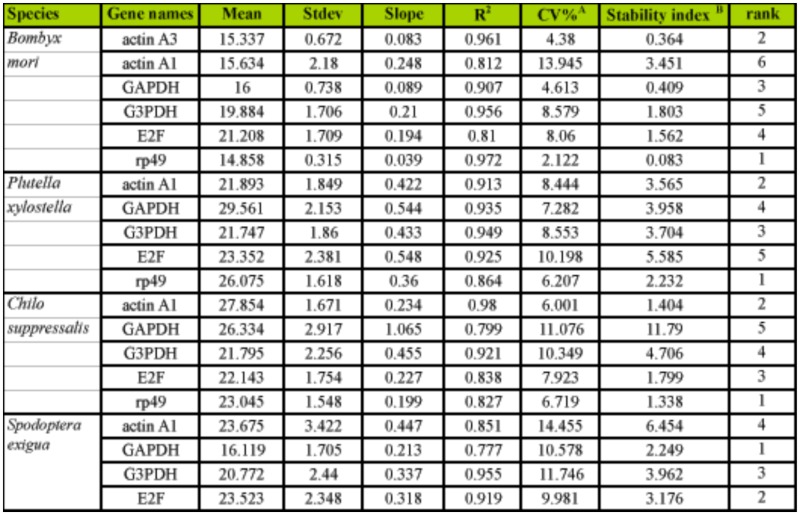
Evaluation of the stability of selected housekeeping genes (HKGs) in all tested samples using stability index assay. HKGs with the lowest stability index are treated as the best control.

**Supplementary Table 2.**
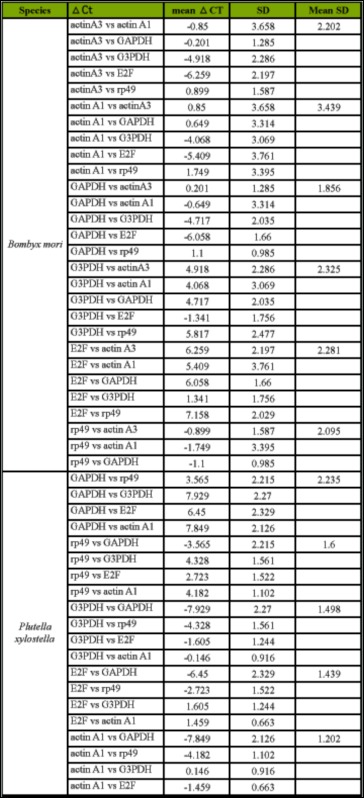
The raw data of gene expression stability analysis by ΔCt approach.

**Continued:**
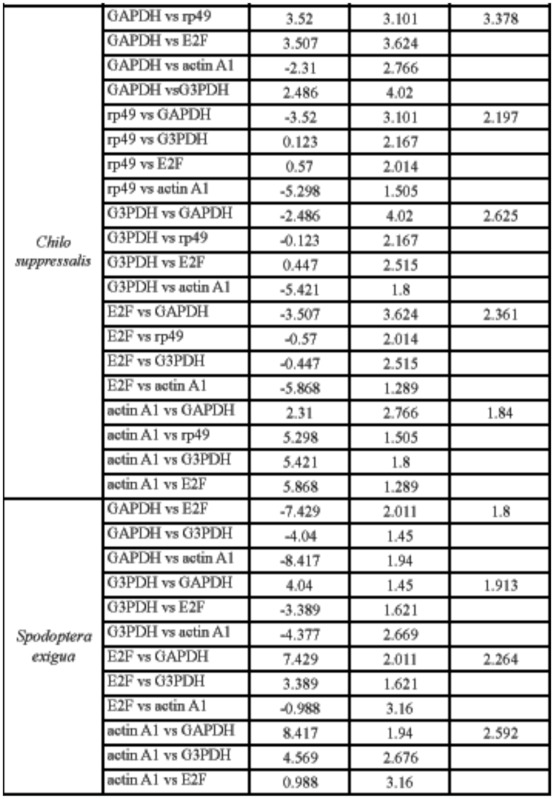

## **Abbreviations: **

qPCR: quantitative real-time polymerase chain reaction
HKGs: housekeeping genes
RT: reverse transcription
Ct: cycle threshold
actin A3: actin gene A3
actin A1: cytoplasmic actin gene A1
GAPDH: glyceraldehyde-3-phosphate dehydrogenase
G3PDH: glycerol-3-phosphate dehydrogenase-2
E2F: E2F transcription factor 4
rp49: ribosomal protein 49
